# Changes in Auditory Evoked Potentials Increase the Chances of Adults Having Central Auditory Processing Disorder

**DOI:** 10.1055/s-0042-1759747

**Published:** 2023-10-06

**Authors:** Andressa Pelaquim, Milaine Dominici Sanfins, Marco Aurélio Fornazieri

**Affiliations:** 1 Postgraduate Program (Doctorate) in Health Sciences, State University of Londrina (UEL), Londrina, PR, Brazil; 2 Department of Clinical Audiology, Center for Advanced Electrophysiology and Neuroaudiology (CENA), Israeli Institute of Education and Research Albert Einstein, São Paulo, SP, Brazil.; 3 Department of Clinical Surgery, Londrina State University (UEL) and Pontifical Catholic University of Paraná, Londrina, PR, Brazil

**Keywords:** electrophysiology, auditory processing disorder, auditory evoked potentials, adults

## Abstract

**Introduction** Auditory evoked potentials are widely used in clinical practice to complement the assessment of central auditory processing. However, it is necessary to understand whether these potentials are highly accurate, to assist in the diagnosis of auditory processing disorder.

**Objective** To measure the accuracy of middle and long latency auditory evoked potentials in the diagnosis of auditory processing disorder in adults.

**Methods** This is a case-control study, formed by a control group of 30 individuals with normal auditory processing assessment, and a case group composed of 43 individuals with altered auditory processing assessment. Their sensitivities, specificities, accuracies, positive and negative predictive values for the diagnosis of alterations were measured and compared between the potentials.

**Results** The accuracies of the middle and long latency potentials were 51% and 67%, respectively. The P1-N1-P2 and N2-P300 complexes had an accuracy of 57.5% and 58.9%, respectively. The cognitive potential P300 showed an accuracy of 55%. There was no significant result for the middle-latency potential (OR = 1.8; 95% CI: 0.6–5.4, *p* > 0.42) and for P300 (OR = 2.63, 95% CI: 0.85–8.43, *p* > 0.11). However, the result was significant for the long-latency potential (OR = 6.3; 95% CI: 2–19.6, *p* < 0.01). There was a significant result for the P1-N1-P2 complexes (OR = 6.76, 95% CI:1.4–32.5, *p* = < 0.010) and N2-P300 (OR = 3.60; 95% CI: 10.16–11.20, *p* < 0.039).

**Conclusion** Individuals with altered long-latency auditory evoked potential are more likely to have auditory processing disorder and, as such, this test can be used as a complementary tool to confirm the diagnosis.

## Introduction

The interpretation of acoustic information is performed by the Central Auditory Nervous System (CANS), through the occurrence of a cascade of mechanisms. For sound information to be detected and interpreted properly, the anatomical and functional integrity of the peripheral and central auditory pathways is necessary, so that the processing takes place effectively. 1 2

Auditory processing (AP) refers to the efficiency and effectiveness with which the CANS uses verbal and nonverbal auditory information. 3 It is widely studied, mainly with the aim of identifying and clarifying the hearing difficulties of children and adults in relation to sound perception, even while having thresholds within the normal range. 4 The AP includes mechanisms underlying the abilities of sound localization and lateralization, auditory discrimination and recognition, temporal aspects of hearing, such as temporal integration and discrimination, temporal ordering and masking, auditory performance in dichotic listening, and performance in degraded acoustic speech signals. 3 5

The assessment of central auditory processing (CAP) consists of checking for one or more altered auditory skills. It consists of behavioral tests capable of identifying Central Auditory Processing Disorder (CAPD). 1 6 The APD refers to a deficit in the neural processing of acoustic stimuli, through preserved cognitive and language skills. However, this disorder can be the cause or coexist with specific alterations in language and learning, among other neurological alterations. 3

The American Speech-Language-Hearing Association (ASHA) 3 recommends that AP assessment be complemented by the electrophysiological assessment, through the Auditory Evoked Potentials (AEPs). The use of middle- and, mainly, long-latency auditory evoked potentials in AP alterations has been studied in recent years. Therefore, it reinforces the need for further studies to establish the clinical utility of AEPs in APD cases.

The AEPs assess the neuroelectric activity of the central auditory pathway, starting in the auditory nerve up to the auditory cortex. 7

The Brainstem Auditory Evoked Potential (ABR) evaluates the electrical activity of the first neurons of the auditory system up to the brainstem. It is the most used AEP. 7 8 The Middle Latency Auditory Evoked Potential (MLAEP) reflects cortical activity related to the primary auditory skills of recognition, discrimination, and figure-ground and non-primary skills, such as selective attention, auditory sequence, and auditory/visual integration. 9 10 Long-Latency Auditory Evoked Potential (LLAEP) is composed of sequential waves P1, N1, P2 and N2. The P1-N1-P2 complex evidences the arrival of the sound stimulus to the auditory cortex and the beginning of cortical processing, being very important to verify if the acoustic signal was received properly. The N2 wave is considered a mixed component related to sound stimulus discrimination. Furthermore, the P300 cognitive component is between 300 and 500 ms post-stimulation. It reflects the activity of cortical auditory areas related to discrimination, integration, and auditory memory skills. 11 12 13

In all age groups, the performance of the AP assessment is consolidated, as well as the use of electrophysiological tests is highly recommended to complement the diagnosis. There are several previous studies in the literature involving the AP and the AEPs, especially regarding the P300 cognitive component. 11 14 15 16 Additionally, there are studies that sought to investigate the electrophysiological activity of the central auditory pathway in cases of APD, correlating the objective findings with the behavioral ones. 17 18 However, there are no in-depth studies on the accuracy of AEPs in AP alterations in adults, without other associated pathologies.

Accuracy is considered in epidemiology a measure of high validity, being widely applied in studies on the evaluation of diagnostic or screening tests. Its investigation makes it possible to verify the degree to which the data measure what they should measure or how much the results of an assessment correspond to the true state of the phenomenon being measured. 19

The aim of this study was to measure the accuracy of the middle- and long-latency auditory evoked potential in adults with central auditory processing disorders.

## Methods

### Participants

This is a case-control study. The sample was recruited through an invitation directed by e-mail to the academic community of State University of Londrina (UEL).

The community was informed about the objective and justification of the study, the inclusion and exclusion criteria, the place where the exams were performed, as well as the researchers' telephone number and email address, in case they were interested in participating. Only individuals without otoscopic alterations, with normal hearing thresholds, according to the criteria of the World Health Organization (WHO) 20 for the adult population, tympanometry with peak of maximum compliance around atmospheric pressure of 0 daPa and equivalent volume of 0.3 and 1.3 ml for both groups, ipsilateral and contralateral stapedial acoustic reflexes present for the control group 21 integrity of the auditory pathway of the brainstem verified by the ABR, with or without complaints of difficulty in understanding speech in silence and in noise, difficulty in auditory memory, and complaint of inattention. Individuals with an otologic history of alteration or pathology in the middle ear, previous diagnosis of type I or II diabetes, neurological or neurodegenerative diseases, previous auditory training for APD intervention, and drug users were excluded.

The present study was approved by the Human Research Ethics Committee, CAAEE: 95467918.2.0000.5231. Data were collected at an audiological clinic specializing in hearing and balance, in the city of Londrina, Paraná, Brazil, between August 2018 and August 2019. All participants were instructed and signed the informed consent (IC) form.

### Study Design

In the first stage, the volunteers underwent a basic audiological assessment to define audibility thresholds and conditions of the middle ear, and a complete CAP exam to identify individuals with altered AP. In the second stage, the electrophysiological assessment was performed, consisting of ABR, MLAEP and LLAEP. The ABR was performed before the other potentials, to verify the integrity of the brainstem auditory pathway.

After the two steps described, the exams were evaluated by an examiner experienced in audiology and the volunteers were divided into two groups. One group consisting of controls ( *n* = 30) with normal hearing thresholds and no changes in the CAP exam and a group of cases ( *n* = 43), composed of individuals with normal auditory thresholds and with alterations in the AP exam.

### Procedures

#### Immitanciometry, Audiometry and Logoaudiometry

Tympanometry was performed using the Otometrics OTOFLEX 100 (Natus Medical Inc., Middleton, WI, USA) equipment and a probe with a 226Hz tone. The ipsilateral and contralateral acoustic reflexes were investigated in both ears at sound frequencies of 500, 1,000, 2,000 and 4,000Hz.

In the pure tone audiometry, a two-channel MADSEN ITERA II (Natus Medical Inc., Middleton, WI, USA) audiometer calibrated to the ANSI-69 standard and TDH39 supra-aural headphones, was used as a stimulus transducer. Hearing thresholds were surveyed via air at frequencies of 250, 500, 1,000, 2,000, 3,000, 4,000, 6,000, and 8,000Hz. The speech audiometry was composed by the speech recognition threshold (SRT), which was performed live through a list of trisyllables and the intensity in which the participant hit 50% of the presented words was adopted as a result. To perform the percentage index of speech recognition (PISR), 30dB were added above the tonal threshold of the average of 500, 1,000, and 2,000Hz. A list of phonetically balanced monosyllabic words was used, which were presented to the individual by means of recording. 22 A percentage of correct answers between 88 and 100% was considered normal.

#### Assessment of Central Auditory Processing

The battery of tests for the CAP assessment consisted of non-verbal stimuli, except for the dichotic digit test, presented through CDs, according to the literature. 23 24 25 The test selection procedures followed the standards suggested by the Clinical Guide. 26 The assessment consisted of the following tests: speech-in-noise (SIN) test, binaural interaction and separation, frequency pattern test (PPS), Random Gap Detection Test (RGDT), and Masking Level Difference (MLD). The normality standard considered for each test was the one proposed in the literature. 23 24 25 27

#### Electrophysiological Assessment

The electrophysiological assessment was performed with the SMART – EP (Intelligent Hearing Systems, Miami, FL, USA) equipment and the Insert ER – 3A transducers (Natus Medical Inc., Middleton, WI, USA), in an acoustically and electrically prepared room. The subjects were accommodated in a reclining chair in a comfortable position. Before starting the collection, the skin of each subject was cleaned using a Nuprep abrasive paste (Weaver and Company, Aurora, CO, USA) in the places where the Solidor disposable electrodes (São Paulo, SP, Brazil) were fixed. Then, they were fixed using the Tem 20 electrolytic paste (Weaver and Company, Aurora, CO, USA) to improve the electrical conductivity.

Subjects were instructed to keep their eyes closed during the assessment to avoid artifacts, while awake. All assessments were performed monaurally under two conditions: assessment of the right ear and assessment of the left ear.

The assembly of the electrodes followed the standards established by the International Electrode System (IES) 10 to 20 for its correct use. The electrode impedance remained below 3 KΩ and the difference between the electrodes was below 2 KΩ for all exams.

#### MLAEP

The electrodes were arranged as follows: ground electrode on the forehead (A); the active (positive) electrodes in the right and left coronal region (C4 and C3); the reference electrodes (negative) on the right and left ear lobes (A2 and A1), using the two channels of the equipment. A jumper was used to connect the inputs of the reference electrodes of channel A and B.

In the acquisition of the MLAEP, two collections were performed containing 1,000 intermediated stimuli and free of artifacts, and the responses were recorded twice in each condition (C3A1, C4A1, C3A2, C4A2) to increase reliability. The components were identified and marked by the researcher, following the baseline. The Na component was the first negative peak identified between 16 and 30ms; Pa was the next highest positive peak observed between 30 and 45ms; Nb was the second negative peak located between 46 and 56ms; and Pb was the second negative peak identified between 55 and 65ms. 28

The functional analysis of the CANS was performed by comparing the interamplitude of Na and Pa between the ears and between the cerebral hemispheres. Each response on one side and the other should not be less than 50% in the same individual. The presence of electrode effect and ear effect configured a functional abnormality of the CANS. 29

#### LLAEP

The active electrodes were positioned at the vertex (Channel A - Cz) and (Channel B - Fpz), the reference electrode at the right (A2) and left lobes (A1) and the ground electrode at Fpz. A jumper was used to connect the inputs of the reference electrodes of channel A and B.

The subjects were instructed to count aloud the number of rare stimuli so that the assessment could be performed correctly. Only the tracing of the rare stimulus captured in Cz in both ears was considered for the analysis and for presenting better morphology in relation to Fz. The collections considered were those with artifact values lower than 10%. The following components were identified and manually marked by the researcher: P1, N1, P2, N2, and P300. The P1 component was identified between 54 and 73ms; N1 was the first negative peak found between 83 and 135ms; P2 was the second positive peak located between 137 and 194ms; and N2 was the second negative peak observed between 188 and 231ms. The P300 cognitive component was the third positive peak identified between 225 and 365ms for individuals between 17 and 30 years, and between 290 and 380ms for individuals between 30 and 50 years. 30 However, the presence of positive double deflection in P300 was verified, to correctly identify the presence of the P3a and P3b component. According to the literature, P3a occurs around 280ms and P3b has latency equal to or above 300ms. Thus, we consider the third positive peak with latency equal to or greater than 300ms as cognitive P300. 30

The parameters for acquiring the MLAEP and LLAEP are described in Table 1 .

**Table 1:** Parameters used to acquire the MLAEP and LLAEP 28 31

Parameters	MLAEP	LLAEP
Stimulated ear	OD / OE	OD / OE
Stimulus type	Click	Nonverbal/tone burst
Presentation Rate	9.8 / sec	1.1 / sec
Number of scans	1,000	300
Polarity	Rarefied	Alternate
Intensity	70dB	75dB
Frequency of frequent stimulus	−	1,000Hz
Percentage of frequent presented stimulus	−	80%
Frequency of rare stimulus	−	2,000Hz
Percentage of rare presented stimulus	−	20%
High pass acquisition filter	20Hz	10Hz
Low pass acquisition filter	1,500Hz	300Hz
High pass analysis filter	10Hz	−
Low pass analysis filter	100Hz	−
Analysis time	70 ms	533 ms

**Abbreviations** : dBNA - decibels; ms - milliseconds; HZ - Hertz; LLAEP- Long latency auditory evoked potentials; MLAEP- Middle Latency Auditory Evoked Potential; OD- Right ear; OE- Left ear.

### Statistical Analysis

The sample was calculated considering a difference in the percentage of presence of alteration in MLAEP and LLAEP of 40% between the group with normal and altered auditory processing. With a significance level of 5% and a power of 80%, the need for 23 individuals per group was determined. An addition of 7 subjects per group was made to increase the accuracy of secondary analyses.

The accuracy of the tests was verified through diagnostic tests of sensitivity, specificity, positive predictive value, and negative predictive value. The chance of change in CAP due to changes in electrophysiological tests was calculated by logistic regression. Categorical variables were analyzed using the Fisher exact test. Furthermore, *p* -values < 0.05 were considered significant. Data were analyzed using the Statistical Package for the Social Sciences (SPSS, IBM Corp. Armonk, NY, USA), version 20.0.

## Results

Among the 147 individuals who agreed to participate, 73 could be included in the study. The control group was composed of 63% of female subjects and the study group of 65% of female subjects. Most participants had completed or ongoing university education, aged 18 to 55 years, of both sexes, with normal hearing thresholds, and the demographic characteristics between volunteers with CAP alteration and controls were matched ( Table 2 ).

**Table 2:** Demographic characteristics of participants by study group

Variables	Categories	Controls ( *n* = 30)	APD ( *n* = 43)
Age (mean, SD)	≥ 18 years ≤ 55 years	29.4 (7.9)	29.3 (6.9)
Sex (%)	Men	36.7	34.8
	Women	63.3	65.2
Race (%)	White	100	100
	Non-White	0	0
Education (%)	< Highschool	0	0
	Highschool	50	27.9
	Undergraduate degree or Higher	50	72.1

**Abbreviations:** APD- auditory processing disorder; SD- standard deviation.

The MLAEP showed low sensitivity and high specificity to detect individuals with AP alterations. It also presented an accuracy of 51.4% for APD cases. Individuals with altered MLAEP were 1.78 times more likely to have APD (odds ratio, OR: 1.78, 95% confidence interval, CI: 0.6–5.4, *p* > 0.42), that is, it is not a good test to aid in the diagnosis of APD ( Table 3 ).

**Table 3:** Comparison between electrophysiological tests in the diagnosis of central auditory processing disorders

Electrophysiological tests	N	Sensitivity	Specificity	PPV	NPV	Accuracy	OR (95% CI)	*p* -value
MLAEP	70	32%	80%	68%	45%	51%	1.78 (0.65.4)	0.42
LLAEP	73	56%	84%	83%	57%	67%	6.3 (2–19.6)	0.01*
P1-N1-P2 Complex	73	32%	93%	87%	49%	57%	6.76 (1.4–32.5)	0.01*
N2-P300	73	42%	83%	78%	50%	58%	3.6 (1.16–11.2)	0.039*
P300	73	35%	83%	75%	49%	55%	2.63 (0.85–8.43)	0.112

**Abbreviations:** APD- Auditory Processing Disorder; CI- Confidence interval; LLAEP- Long latency auditory evoked potentials; MLAEP- Middle Latency Auditory Evoked Potential; N- number of individuals; NPV- Negative Predictive Value; OR- Odds Ratio; P300 event related potential; PPV- Positive Predictive Value. **Notes:** The LLAEP, as well as the analysis of its complexes, demonstrated high accuracy in the detection of neurophysiological alterations, at the level of the primary auditory cortex, in adult individuals with APD. *p* -value <0.05; Fisher exact test; * Significant.

The LLAEP, as well as the subcomponents P1-N1-P2 and N2-P300, demonstrated showed low sensitivity and high specificity. The accuracy of the LLAEP encompassing all components was 67.1%, a 15.7% higher rate than the MLAEP. Individuals with altered LLAEP were six times more likely to have APD, which confirms that it is a good exam to complement the diagnosis (OR = 6.3, 95% CI: 2–19.6, *p* < 0.01) ( Table 3 ).

The P1-N1-P2 complex accuracy was 57.5%. Individuals with alterations in this complex were six times more likely to have APD (OR = 6.76, 95% CI: 1.4–32.5, *p* < 0.010). The N2-P300 complex obtained an accuracy of 58.9%. Individuals with altered N2-P300 were three times more likely to have the AP test altered (OR = 3.60; 95% CI: 1.16–11.20, *p* < 0.039). Finally, the cognitive component P300 did not obtain significant results, presenting an accuracy of 55% (OR = 2.63; 95% CI: 0.85–8.43, *p* > 0.11) ( Table 3 ).

Secondary results ( *supplementary material* ).

## Discussion

The present study demonstrates that individuals with altered LLAEP are six times more likely to have APD. Thus, the LLAEP is an efficient electrophysiological method to be associated and used to confirm the diagnosis of APD ( Table 3 ). However, due to their low sensitivity, we emphasize that electrophysiological tests should not be used alone, as a screening or diagnostic method, in adult individuals with APD complaints.

In our study, we observed that among all the analyses, the MLAEP was the one with the lowest accuracy ( Table 3 ), indicating that it is not a good evaluation method to aid in the diagnosis of APD (51%, OR = 1.78). The low accuracy found in our study may justify a previous study that did not observe a correlation between the AEPs and temporal pattern tests 32 and others that identified a weak and moderate correlation between the results of the MLAEP and the behavioral tests of the AP, respectively. 17 18

Regarding the analysis of the latency of the Pa component and the interamplitude of Na-Pa, we did not observe any difference between the group with and without APD, considering the leads C3A1/C3A2/C4A2/C4A1 ( Supplementary Tables S1 and S2 , *supplementary material* ). However, we numerically observed a decrease in Na-Pa interamplitude in individuals with APD in our data, in line with a study 33 that observed lower latencies of the Na and Pa components, as well as the Na-Pa interamplitude for individuals with APD compared with controls. Perhaps, a larger sample could have statistically demonstrated this difference.

The analysis of the Pa wave amplitude demonstrates the presence of the electrode effect and/or ear effect. Presence indicates alteration and is one of the main ways of evaluating the results of the MLAEP. 32 In our results, there was the presence of the ear effect ( Supplementary Figure S1 , *supplementary material* ), consistent with a study that concluded that the presence of the ear effect is more compatible with cases of APD, to the detriment of the presence of the electrode effect, which is more evident in cases of neurological injuries. 34 When evaluating three different cut-off points (50, 40, and 30%), we found that the lower the cut-off point, the greater the percentage of altered MLAEP ( Supplementary Figure S2 , *supplementary material* ). This finding corroborates a study that demonstrated that the 30% cutoff point is more reliable to identify neurological lesions and APD. 34

The LLAEP, as well as the P1-N1-P2 and N2-P300 complexes analyzed separately, showed good accuracy, in relation to MLAEP ( Table 3 ). This finding justifies the use of LLAEP in studies with different populations. Kumar et al. 35 observed higher latencies and reduced amplitudes of P1, N1, and P2 components in individuals with type II diabetes. Oliveira et al. 36 identified a relationship between LLAEP and cognitive performance in the elderly population. Prestes et al. 37 identified that adults who stutter have worse performance in auditory temporal processing skills and increased latencies of the N2 and P300 components. In addition to these, another study identified alterations in LLAEP components in children with APD. 38 Our results also demonstrated that the potential has good specificity, both for the joint analysis of all components and for the analysis of the complexes ( Table 3 ). Furthermore, by evaluating the latency values of the LLAEP components ( Supplementary Table S3 , *supplementary material* ), we identified the increase for N1, P2, and N2, in subjects diagnosed with APD. As for the amplitudes, we observed numerically smaller amplitudes for individuals with APD ( Supplementary Table S4 , *supplementary material* ). The increase in latency and decrease in the amplitude of the components is expected in cases of APD, as a neurobiological alteration is observed in the SNAC, which directly affects the auditory abilities. 39

Given the above, we can say that the LLAEP is the best electrophysiological method to assess CANS at the cortical level, and thus complement the assessment of the AP.

Regarding the P300 cognitive potential, our results indicated that it does not show good accuracy for APD cases ( Table 3 ). The test is often performed in clinical routine for the evaluation of AP, especially in school-age children and adolescents who may or may not have other pathologies or associated complaints. 18 40 41 42 Considering P300 captures the potentials related to the executive functions of memory and attention, in our study, we excluded complaints phonological, reading, writing difficulties, among others. That could influence the AP assessment. Thus, even having presented specificity above 80%, the chance of an individual with APD having altered P300 was not significant.

It is important to emphasize that there is a lot of variation in the results in the literature, usually due to the protocol used, small sample sizes, and the form of analysis used, which are mostly correlations or just descriptions of results through cases. Additionally, it was found that studies on auditory processing are mostly performed with school-age children and adolescents 43 44 with reading and writing difficulties, learning 45 phonological alterations, and associated pathologies, such as attention deficit, dyslexia, and autism. 46 47 Thus, we point out the difficulty of finding studies with samples composed only of adults with characteristic complaints of APD to compare with our results. Another point is the fact that the studies did not use behavioral tests with a low linguistic load as a protocol for execution or did not use the complete minimum protocol recommended by ASHA. 3

The present study has some limitations. First, it was performed only with adult subjects. There are few studies involving AP and electrophysiological assessment in adults with normal hearing thresholds in the literature. One hypothesis would be the lack of knowledge about the AP and its abnormalities, and consequently the nonidentification of changes, in addition to the ability to create strategies to address the complaints. This hypothesis is consistent since in our study most the adult individuals were university students or had already graduated and not had complaints, but had alterations in the AP exam, which made it difficult to find a healthy individual. Additionally, it was not easy to obtain a homogeneous sample regarding gender, to perform an analysis separately, since there was little male adherence to the research.

Another relevant point is the type of stimulus used. We used the click stimulus in MLAEP and the oddball stimulus for LLAEP since we wanted to eliminate the interference of speech processing in the results. Nevertheless, it is important to point out that speech stimuli are already used in the assessment of LLAEP and P300 because they are more complex to be processed through the auditory pathway. 48

## Conclusion

Individuals with altered LLAEP were more likely to have APD and, therefore, the test can be used as a complementary tool to confirm the diagnosis. The MLAEP did not prove to be a good test to aid in the diagnosis of APD in adults.
