# Supplementary material for: Changes in Auditory Evoked Potentials Increase the Chances of Adults Having Central Auditory Processing Disorder
Source: Int Arch Otorhinolaryngol. 2023 Oct 6;28(1):e134–40. doi: 10.1055/s-0042-1759747 (PMC10843927; doi:10.1055/s-0042-1759747)
Supplement: Supplementary file 1 — Supplementary Material [file 10-1055-s-0042-1759747-s221342.pdf]

Supplemental Information

Results

Secondary Analysis

The MLAEP analysis showed numerically that individuals with APD presented a decrease in the Na-Pa interamplitude, indicating a possible decrease in synaptic activity in the subcortical and thalamic regions. However, there was no difference in the mean latency of the Pa component between individuals with and without APD, as well as for the Na-Pa interamplitude in leads C3A1, C4A1, C3A2, and C4A2. (►**Supplementary Tables S1** and **S2**).

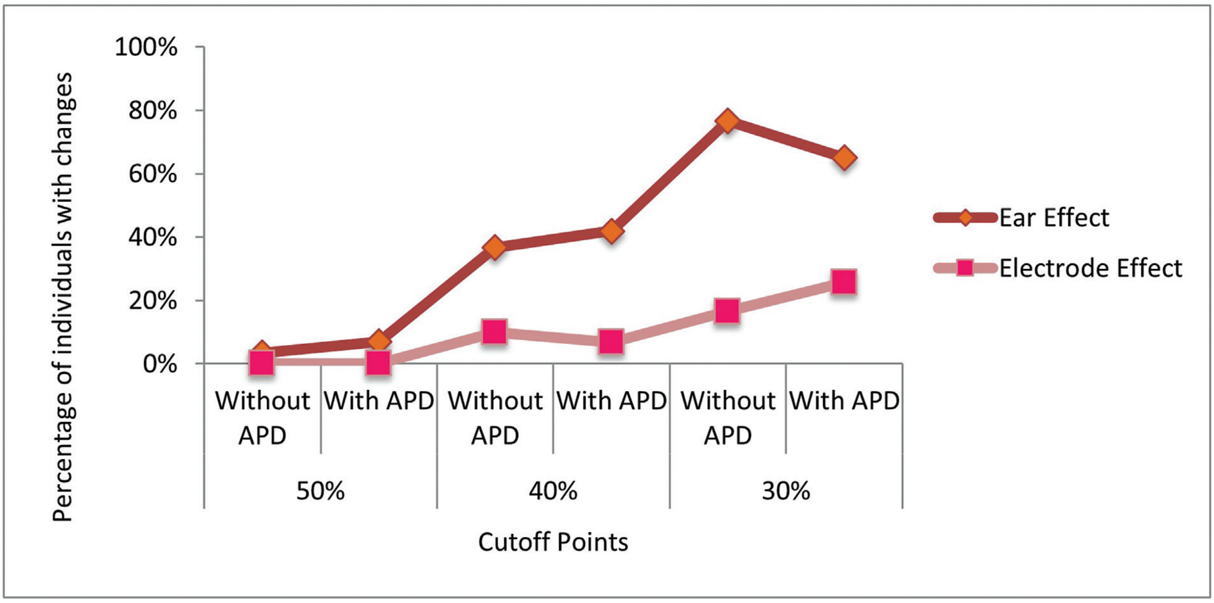

Supplementary Fig. S1 Percentage of Electrode Effect and Ear Effect according to the cutoff points of 50, 40, and 30%. **Legend:** Smaller cut-off points tend to be more sensitive to detecting changes. Additionally, the predominance of the ear effect is observed in individuals with APD. APD = Auditory processing disorder.

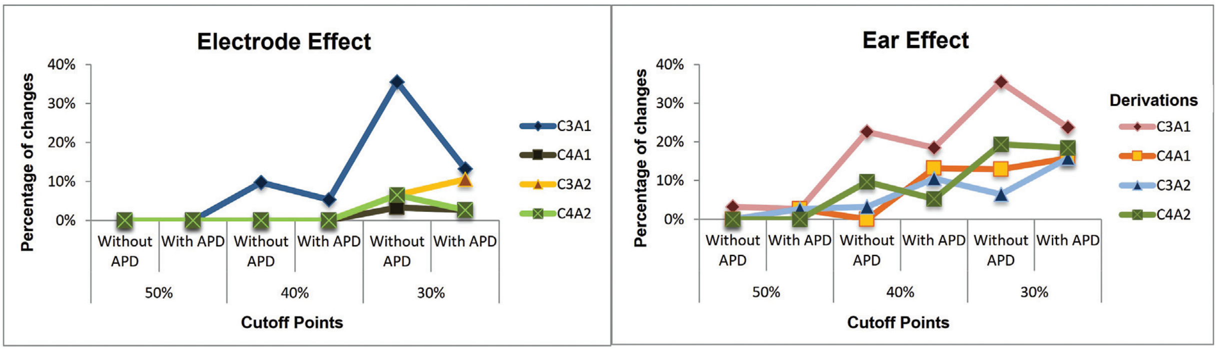

Supplementary Fig. S2 Presence of Electrode Effect and Ear Effect according to the derivations and cutoff points of 50, 40 and 30%. **Legend:** The cut-off point of 30% proved to be more sensitive to the presence of electrode effect and ear effect. It is observed that the presence of the ear effect was predominant in the sample, for all the analyzed leads. This result confirms that the presence of the ear effect is more compatible with APD, and that MLAEP is a good method for identifying and evaluating individuals with brain injuries. APD = Auditory processing disorder.

 © 2024. Fundação Otorrinolaringologia. All rights reserved.

Supplementary Table S1 Comparison between the interamplitude values of the Na-Pa (cut 50%) in adult individuals with and without APD

Interamplitude	Derivations							
	C3A1 Na-Pa		C4A1 Na-Pa		C3A2 Na-Pa		C4A2 Na-Pa	
microvolt (μV)	Without APD	With APD	Without APD	With APD	Without APD	With APD	Without APD	With APD
Mean	1.07	0.9	1.23	1.02	1.16	0.99	1.19	1.03
Median	0.78	0.83	1.04	1.04	1.05	0.92	1.16	1.1
SD	0.92	0.34	0.73	0.29	0.76	0.38	0.69	0.35
IQR	0.52	0.48	0.55	0.45	0.52	0.54	0.83	0.47
p-value	0.872*		0.517*		0.533*		0.426*	

Abbreviations: APD- Auditory processing disorder; IQR- Interquartile Range; SD- Standard Deviation; Microvolt
Notes: *p*-value < 0.05; Mann-Whitney Test*; Student T-Test**.

Supplementary Table S2 Comparison between the latency values of the Pa component in adult individuals without APD and with APD

Component	Derivations							
	C3A1 Pa		C4A1 Pa		C3A2 Pa		C4A2 Pa	
	Without APD	With APD	Without APD	With APD	Without APD	With APD	Without APD	With APD
Mean (Ms)	29.23	29.51	29.95	29.95	28.08	28.06	28.31	28.35
Median	29.5	29.3	29.80	29.9	28.2	28.1	28.9	28.7
SD	3.39	4.02	3.59	3.74	3.86	3.9	3.35	3.76
IQR	2.8	3.9	4.0	4.0	3.4	4.4	3.7	4.4
p-value	0.734*		0.941*		0.981**		0.963**	

Abbreviations: APD- Auditory processing disorder; IQR- Interquartile Range; SD- Standard Deviation; ms - miliseconds.
Notes: *p*-value < 0.05; Mann-Whitney Test*; Student T-Test**.

The investigation of the presence of electrode effect and/or ear effect indicated a greater presence of ear effect in both groups, considering the three different cutoff points 50, 40 and 30%. Furthermore, the results showed that cutoff points lower than 50% tended to increase the rate of altered exams, even in individuals without CAPD (►**Supplementary Figure S1**). As for the analysis by leads, it was observed that the ipsilateral leads (C3A1 and C4A2) tended to present a higher rate of alteration for the ear effect (►**Supplementary Figure S2**).

When comparing the latencies of components P1, N1, P2, N2 and P300 between the groups with and without CAPD, individuals with CAPD had higher latencies in both ears (►**Supplementary Table S3**). As for the amplitude values, the P1 component in the right ear presented a difference between the groups, and it was observed that the amplitudes of the N2 and P300 components were lower in the group of individuals with CAPD. (►**Supplementary Table S4**).

Supplementary Table S3 Comparison between the latency values of the components of LLAEPs in adult individuals without APD and with APD

Components	Mean (ms)	Median (ms)	SD	IQR	*p*-value
P1 RE<br>Without APD<br>With APD	56.13<br>55.5	54.0<br>49.0	15.47<br>17.52	28<br>26	0.688*
P1 LE<br>Without APD<br>With APD	50.77<br>57.71	49.0<br>56.0	13.14<br>18.56	13<br>34	0.066**

(*Continued*)

 © 2024. Fundação Otorrinolaringologia. All rights reserved.

Supplementary Table S3 (*Continued*)

Components	Mean (ms)	Median (ms)	SD	IQR	*p*-value
**N1 RE**<br>**Without APD**<br>**With APD**	105.42<br>114.86	107.0<br>112.5	16.11<br>18.11	26<br>20	**0.04***
**N1 LE**<br>**Without APD**<br>**With APD**	103.53<br>113.33	104.0<br>110.5	22.47<br>18.99	31<br>26	**0.047****
**P2 RE**<br>**Without APD**<br>**With APD**	167.77<br>179.19	163.0<br>175.5	33.72<br>26.46	23<br>32	**0.012***
**P2 LE**<br>**Without APD**<br>**With APD**	162.03<br>178.62	161.0<br>179.5	29.87<br>27.21	28<br>33	**0.016****
**N2 RE**<br>**Without APD**<br>**With APD**	206.58<br>223.31	201.0<br>219.0	31.49<br>26.97	29<br>27	**0.001***
**N2 LE**<br>**Without APD**<br>**With APD**	199.61<br>219.05	194.0<br>215.5	32.99<br>25.53	32<br>31	**0.001***
**P300 RE**<br>**Without APD**<br>**With APD**	286.71<br>291.48	320.0<br>314.5	97.68<br>99.21	35<br>46	0.596*
**P300 LE**<br>**Without APD**<br>**With APD**	267.84<br>273.81	304.0<br>318.5	106.9<br>115.66	41<br>59	0.136*

Abbreviations: APD- Auditory processing disorder; IQR- Interquartile Range; LE- Left Ear; RE- Right Ear; SD- Standard Deviation.
Notes: *p*-value < 0.05; Mann-Whitney Test*; Student T-Test**; *Significant.

Supplementary Table S4 Comparison between the amplitude values of the components of LLAEPs in adult individuals without APD and with APD

Component Amplitudes	Mean (ms)	Median (ms)	SD	IQR	*p*-value
**P1 RE**<br>**Without APD**<br>**With APD**	2.95<br>3.91	3.2<br>3.77	1.65<br>2.05	2.41<br>2.88	**0.036****
**P1 LE**<br>**Without APD**<br>**With APD**	3.72<br>3.35	3.69<br>3.09	2.09<br>1.82	2.9<br>2.57	0.424**
**N1 RE**<br>**Without APD**<br>**With APD**	4.97<br>5.84	5.04<br>5.56	3.5<br>3.39	3.7<br>4.7	0.222*
**N1 LE**<br>**Without APD**<br>**With APD**	4.33<br>4.53	3.91<br>3.99	3.28<br>2.63	3.24<br>2.21	0.479*
**P2 RE**<br>**Without APD**<br>**With APD**	2.26<br>2.57	1.59<br>2.37	2.1<br>2.24	2.29<br>3.64	0.600*
**P2 LE**<br>**Without APD**<br>**With APD**	1.87<br>2.47	1.5<br>2.16	1.67<br>1.89	3.09<br>2.8	0.145*
**N2 RE**<br>**Without APD**<br>**With APD**	9.17<br>8.26	8.68<br>7.6	5.23<br>5.9	8.06<br>6.24	0.375*

 © 2024. Fundação Otorrinolaringologia. All rights reserved.

Supplementary Table S4 (*Continued*)

Component Amplitudes	Mean (ms)	Median (ms)	SD	IQR	*p*-value
**N2 LE**					
**Without APD**	9.76	7.5	6.55	8.82	0.326*
**With APD**	8.25	7.34	5.85	7.61	
**P300 RE**					
**Without APD**	9.0	8.68	5.45	8.01	0.360*
**With APD**	8.06	7.5	6.04	6.3	
**P300 LE**					
**Without APD**	9.24	7.49	6.92	9.35	0.434*
**With APD**	7.91	7.34	6.21	7.72	

Abbreviations: APD- Auditory processing disorder; IQR- Interquartile Range; LE- Left Ear; RE- Right Ear; SD- Standard Deviation.
Notes: *p*-value < 0.05; Mann-Whitney Test *; Student T-Test**; *Significant.

 © 2024. Fundação Otorrinolaringologia. All rights reserved.
